# Research strategies in nutrition in health and disease: The failure of mechanistic research

**DOI:** 10.3389/fnut.2023.1082182

**Published:** 2023-01-19

**Authors:** Norman J. Temple

**Affiliations:** Centre for Science, Athabasca University, Athabasca, AB, Canada

**Keywords:** cohort studies, randomized controlled trials, mechanistic research, coronary heart disease, saturated fatty acids, cancer, drug discoveries, reductionism

## Abstract

This paper critically evaluates different research methods in order to assess their value for establishing which dietary changes are most effective for protecting health and preventing disease. The evidence demonstrates that the combined use of observational studies (mainly cohort studies) and randomized controlled trials (RCTs) is the most successful strategy. Studies of the details of body mechanisms in health and disease (mechanistic research) is another commonly used research strategy. However, much evidence demonstrates that it is a far less successful strategy. In order to support the above conclusions research studies from the following areas are discussed: obesity and dietary fat; heart disease and saturated fat; the Mediterranean diet and cardiovascular disease; type 2 diabetes and dietary fiber; and cancer and micronutrients. While mechanistic research has a poor track record in nutrition, it has achieved some success in other areas of biomedical science. This is shown by examining the role of mechanistic research in the discovery of new drugs.

## Introduction

A variety of methods are used in research studies in the area of nutrition in health and disease (1). Commonly used approaches include cohort studies, randomized controlled trials (RCTs), and studies of disease mechanisms. This paper critically evaluates the degree of success of different research strategies. The main focus is the serious limitations of mechanistic research.

Mechanistic research is widely viewed as a foundation of the entire biomedical research enterprise. Many thousands of research studies are carried out each year that investigate the details of body mechanisms in health and disease. This research strategy should—at least in theory—help to reveal the full details of normal body functioning. Furthermore, this research should help explain the malfunctions that occur in disease. If successful, this should lead to a fuller understanding of the causes of different diseases and how they can be prevented and treated. This strategy may be likened to a car mechanic repairing a car that has broken down. She fully understands how cars work, she diagnoses the problem, and then applies her knowledge and skills to fixing the problem.

Reductionism is a term that is closely related to mechanistic research. It is often used in nutrition in reference to the study of individual substances present in food rather than to whole foods. The term mechanistic research is used here as it related more closely to the theme of this paper.

## Research strategies in nutrition in health and disease

When we critically evaluate the contribution of different research methods to our present knowledge of the role of nutrition in health and the prevention disease, we find that the great bulk of our knowledge of practical value has come from observational studies and RCTs. Mechanistic research, in marked contrast, has played a quite minor role (2–4). Several examples are presented to demonstrate the strength of the supporting evidence. The arguments expressed here have rarely been stated in previous papers. Campbell is one of the very small group of nutrition scientists to have stated similar views (5).

### Obesity: How important is dietary fat?

The first example considers the case of dietary fat and obesity, but an equally strong case can be made in relation to other components of the diet, such as dietary fiber and sugar, and their role in the control of body weight.

There has been great interest for many years in the relationship between dietary fat and body weight. Fat has a much higher energy content (9 kcal/g) than either carbohydrate or protein (4 kcal/g). Resultingly, foods with a high fat content also have a high energy density. This, it was widely assumed, means that a fat-rich diet is likely to cause excessive energy intake and thence weight gain. However, there is little solid evidence that supports this view.

Cohort studies have failed to show any clear and consistent association between eating larger amounts of fat-rich foods and increased weight gain (6, 7). For example, meat is associated with risk of overweight but it seems to make little difference if the meat is fatty or lean. Low-fat and fat-free milk show no association with risk of weight gain but neither does whole milk. Nuts are a fat-rich food but are not associated with weight gain.

Dozens of RCTs have been carried out in which subjects in one intervention group have been given a low-fat diet. Some of these studies had the goal of achieving weight loss, some aimed for weight maintenance, while other studies had a goal unrelated to weight. Overall, in those studies with a duration of at least 1 year no evidence emerged that a low-fat diet leads to increased weight loss (8). The findings from RCTs are consistent with those from cohort studies in showing very little association between the quantity of fat in the diet and change in body weight.

Many thousands of studies have examined the mechanisms involved in weight control. This includes such topics as the action of various hormones, the details of intermediary metabolism, and the metabolic effects of different macronutrients. This mechanistic research has told us remarkably little regarding the relationship between dietary fat and body weight. By contrast, the evidence described above compels the conclusion that the overwhelming majority of information of practical value has come from cohort studies and RCTs.

### Saturated fat and coronary heart disease

The relationship between dietary fat and coronary heart disease (CHD) has been investigated for decades. During this time many authoritative statements have been made only to be later shown to be based on flawed evidence. In this respect the story resembles the relationship between dietary fat and body weight as described above.

Changing ideas regarding the relationship between diet and CHD were recently reviewed by Temple (9). For many years it was widely accepted that the leading dietary factor involved in CHD was an excessive intake of saturated fatty acids (SFA). This was based on two sets of observations: first, that SFA causes an increase in the blood cholesterol level (i.e., total cholesterol, TC), and, second, that a high blood TC level is a major risk factor for CHD. It was also shown that polyunsaturated fatty acids (PUFA) lower the TC. Based on these findings it was assumed that replacing SFA with PUFA will lead to a significant reduction in the risk of CHD. Several large RCTs were carried out and these were generally interpreted as supporting the hypothesis. This viewpoint became widely accepted starting around 1974. However, by about 2014 serious flaws with the hypothesis had become impossible to ignore.

Of most importance the findings from cohort studies revealed that the intake of SFA has only a weak association with the risk of CHD. A much stronger association is seen with other components of the diet, especially sugar-sweetened beverages and trans fats (which increase risk), and with whole grains/cereal fiber, fish, and fruit and vegetables (which decrease risk). This leads to the conclusion that SFA plays a much lesser role in the causation of CHD than do several other aspects of the diet.

As noted above the original hypothesis linking SFA with risk of CHD was based on the effect of SFA on the blood TC level. But later research revealed that the strongest indicator of risk is seen for the ratio of the blood TC level to HDL-cholesterol. There are important differences between the effect of SFA (and of other aspects of the diet) on these different types of blood lipids. These differences help explain why SFA does not have a strong association with risk of CHD (9). Note: These measures of blood lipids are included together with cohort studies and RCTs as they have a well-established relationship with risk of CHD. In the case of biomarkers where the association with risk of CHD (or other diseases discussed in this paper) has not been established, the studies are classed as mechanistic research.

The studies referred to above were mainly of two types: (1) cohort studies that investigated the relationship between diet and of various blood lipids on the risk of CHD, and (2) RCTs including clinical trials (the effect of changes in the diet on risk of CHD) as well as studies of how the diet affects the blood level of various lipids. This brief summary of research on diet and CHD reveals that it has taken more than 60 years (from the early studies in the 1950s to the present) to gain a reasonably solid understanding of the subject. Is this an unacceptably long period of time? Was the enormous expenditure of research dollars money well spent?

The only alternative to the types of research study described above is mechanistic research. Over the last several decades tens of thousands of studies have been carried out on the many pathways by which various components of the diet affect the functioning of the coronary artery and other blood vessels. This pied piper research is rich with promises but has failed to deliver. In brief, it does not generate credible information regarding whether SFA and other components of the diet increase or decrease the risk CHD. The conclusion is clear: cohort studies and RCTs (not mechanistic research) is the only research strategy that can reveal what dietary changes are needed in order to prevent CHD.

### Mediterranean diet and cardiovascular disease

Strong evidence from both cohort studies and case-control studies demonstrates a protective association between adherence to a Mediterranean diet (MD) and reduced risk of cardiovascular disease (CVD) (10). As yet, these findings have not been confirmed in RCTs. A valid design requires that groups of subjects are given either a MD or a comparison diet. The PREDIMED study is often characterized as being a demonstration that feeding subjects with a MD leads to a decrease in risk of CVD. However, the major dietary change in the intervention groups was the addition of either extravirgin olive oil or nuts (9). For that reason the PREDIMED study was not a valid trial to test the effectiveness of the MD diet.

The decrease in risk of CVD brought about by the MD can be credited, at least in part, to small reductions in risk factors for CVD, including blood pressure, blood glucose, and waist circumference. However, only small changes are seen in blood lipids (11).

As argued above, mechanistic research has failed to reveal the relationship between SFA and CHD. Mechanisms involving the MD are likely to be far more complex than is the case with SFA as large numbers of different substances are involved. This leads to the conclusion that there is virtually no chance that mechanistic research could furnish solid evidence that the MD prevents CVD.

### Type 2 diabetes and dietary fiber

Cohort studies have repeatedly shown a negative association between the intake of cereal fiber and risk of type 2 diabetes. This was demonstrated most clearly in an umbrella review of meta-analyses that was carried out by Neuenschwander et al. (12). They reported a hazard ratio (HR) of 0.75 (95% CI: 0.65–0.86) when comparing high vs. low intake of cereal fiber. Whole grains had a slightly weaker inverse association (HR of 0.87). A weak association was seen for both vegetable fiber and fruit fiber (HR of 0.93 and 0.95, respectively; not significant).

The large majority of the cohort studies in the above review adjusted for major confounding factors including smoking, body mass index, and physical activity. However, the actual protective substances in whole grains are not known and might include phytochemicals, vitamins, and minerals. Accordingly, it is more scientifically accurate to say that these studies indicate that *foods rich in cereal fiber appear to prevent diabetes* rather than jumping to the conclusion that it is cereal fiber that is largely or solely responsible.

The author is not aware of any RCTs that have tested whether diets high in different types of fiber, as a single dietary change, affect the risk of diabetes. However, RCTs have investigated the effect of whole grains and of cereal fiber on intermediate risk factors, such as insulin sensitivity and the concentration of fasting insulin and C-reactive protein (a marker for inflammation). Such studies should be best characterized as belonging to mechanistic research.

Type 2 diabetes, like the other disorders discussed here, has a complex etiology (13). This leads to the inescapable conclusion that mechanistic research is an extremely inefficient way to discover effective ways to modify the diet in order to reduce risk. Meanwhile, cohort studies have already generated a wealth of reliable information that can be applied to the prevention of diabetes.

### Cancer and micronutrients

Over the last half century many thousands of researchers have striven toward the goal of understanding the mechanisms that lead to the development of cancer. Many billions of dollars have been spent on this global research effort. And what has this mechanistic research achieved? With respect to the area of diet and cancer an appropriate measure of success is that researchers should be able to accurately predict that if the diet is modified in a particular way, this will affect a particular pathway and thereby help prevent cancer. But there is little or no evidence of success for this research strategy.

The failure of mechanistic research in the area of diet and cancer should be contrasted with the many successes of observational studies. Examples include a solid understanding of how the risk of cancer is affected by obesity and the intake of alcohol, processed meat, and fruit and vegetables. Similarly, observational studies have generated a wealth of valuable information regarding the impact of tobacco and exercise on the risk of cancer. As documented below RCTs have also provided a great deal of valuable information.

We now look at several examples from the area of diet and cancer that demonstrate the value of observational studies and RCTs and the weak value of mechanistic research. Population comparisons (also known as ecological studies) that were carried out in the 1970s strongly suggested that dietary selenium is protective against cancer (14). The findings from cohort studies added to this evidence (15). Several RCTs have been carried out. While the findings lack consistency, a plausible interpretation is that selenium supplements prevent cancer in subjects with a low baseline intake of selenium but not in persons with a relatively high intake (16). Research into the mode of action of selenium at the cellular level (i.e., mechanistic research) has identified several possible mechanisms by which the mineral may enhance the prevention of cancer (17). But the complexity of these actions make it extremely difficult to see how this work contributes information of practical value (e.g., being able to predict that an increased intake of selenium, at a particular dose, will prevent one or more types of cancer).

The conclusion that emanates from the findings of research on selenium is repeated with other substances. The focus here is on beta-carotene and vitamin D. Many cohort and case-control studies, mostly carried out in the 1980s, reported an inverse relationship between dietary intake or blood level of beta-carotene and risk of cancer (18). These encouraging reports suggested that beta-carotene prevents cancer. This possibility was tested in several RCTs. But contrary to the outcome that many researchers had hopefully expected, supplements of beta-carotene yielded no evidence of a reduction in risk of cancer (19). There has been much debate regarding the explanation for these seemingly contradictory results. The most plausible explanation for the negative association between the dietary intake of beta-carotene and risk of cancer is that the nutrient is merely a marker for a relatively high intake of fruit and vegetables. In other words, this is an example of confounding.

A similar story has taken place in relation to vitamin D. Various observational evidence suggests that vitamin D may be effective in the prevention of cancer. In particular, prospective cohort studies reveal an inverse association between the blood level of vitamin D and cancer (20). However, the association is neither strong nor consistent. Other investigators carried out ecological studies. Exposure to solar radiation was used as an indirect measure of vitamin D status. Here again, an inverse association was seen for risk of cancer (23). Following these reports RCTs were carried out but the results failed to demonstrate that supplements of vitamin D have any value in the prevention of cancer (21). (Note: the RCTs were also intended to test whether supplements of vitamin D prevent cardiovascular disease and lead to enhanced bone health).

It is clear that the research carried out on beta-carotene, both the observational studies and the RCTs, has been fruitless. In the case of vitamin D the findings are inconsistent: while the observational studies indicate a protective association, the findings from RCTs failed to detect a benefit. But it would be a serious mistake to cast doubt on the value of the research strategy based on using observational studies (mainly cohort studies) in combination with RCTs. Indeed, we can confidently state that if there are nutrients, phytochemicals, or other substances present in food that are effective in the prevention of cancer, then this research strategy is by far the most efficient tool for the identification of those substances. The observational studies and RCTs on beta-carotene and vitamin D should be seen in this context (i.e., the studies on those substances were not failures but were essential steps on the road to eventual success).

Let us now contrast this strategy with mechanistic research. Based on a substantial body of research, multiple different mechanisms have been suggested by which beta-carotene (22) and vitamin D (21) may achieve an anticarcinogenic action. But, as in the case of selenium, the great complexity of the etiology of cancer means we cannot translate these suggestions into practical advice. In other words, we cannot state with any confidence whether an increased intake of either nutrient will decrease the risk of cancer.

## How successful is mechanistic research as a research strategy?

The examples presented above point to the conclusion that mechanistic research is a failed strategy when it comes to discovering how best to improve the diet in order to maintain health and prevent disease. The explanation for this is that the human body is enormously complex and, as a result, it is extraordinarily difficult to properly understand how the factors related to lifestyle, especially diet, affect the pathways that lead to disease. Compounding this, foods contain enormous numbers of different substances. As a result there are vast numbers of possible interactions between food components and body processes. To summarize, investigating how the components of food affect body functioning, such as intermediary metabolism, cellular function, the role of oxidative stress and inflammation, and the actions of the colon microbiome, and then translating this into practical nutritional advice on preventing or treating disease is a strategy that is very unlikely to achieve significant success.

While mechanistic research has a poor record in nutrition, it has achieved success, at least to some extent, in other areas of biomedical science. We can illustrate this by looking at its role in drug discoveries. Mechanistic research has been of crucial importance in the discovery of a variety of important drugs including the following examples: statins (23), beta-blockers (24), calcium channel blockers (25), oral contraceptives (26), drugs for the treatment of depression (serotonin selective reuptake inhibitors [SSRIs] and other drugs) (27, 28), and antiviral drugs (29, 30). However, many drugs owe their discovery more to chance observations than to design that was based on mechanistic research. Prasad and colleagues (31) discussed the history of several of the most common drugs used in cancer chemotherapy. They concluded that: “…rational drug discovery and targeted therapies have minimal roles in drug discovery; serendipity and coincidence have played and continue to play major roles.” Other examples include aspirin, acetaminophen, penicillin, nitroglycerin, sulfonylurea, metformin, thiazides (32), and benzodiazepines (33). In some of these cases the original research involved mechanistic research with the goal of designing a drug for a specific purpose, but, later, as a result of chance findings, the drug was found to be of value for an unrelated purpose. Clearly, both serendipity and mechanistic research have been of crucial importance in drug discovery.

## Discussion

The evidence presented here leads to the conclusion that mechanistic research is of little value as a tool for determining which dietary changes are effective in protecting health and preventing disease.

As mentioned in the Introduction reductionism is a term that is closely related to mechanistic research. The term is often used in nutrition with reference to the study of individual substances present in food rather than to actual foods. Many nutrition researchers have argued that the focus on individual substances has a poor record of success in delivering valuable information in the area of diet and health (34). It is now increasingly argued that the focus of nutrition research should be shifted to the actual foods eaten and dietary patterns. This is because the relationship between diet and health can only be understood as the combined action of the many different substances present in food. This concept is often referred to as food synergy (35, 36).

While mechanistic research has achieved remarkably little in the area of nutrition, a different picture emerges when we look at research in other areas of biomedical science. Of particular note it has led to valuable advances in the search for new and effective drugs. What is the explanation for this? To answer this question we return to the analogy of a car mechanic who repairs a car that has broken down. In order to do that he must understand how cars work, be able to diagnose problems, and then apply his knowledge and skills to fixing the problem. But this is only possible in relatively simple systems such as a car. Drug discoveries can often be analogous to this. In such situations mechanistic research can achieve success. This can occur where a pharmacologist has a solid understanding of a body function and can then apply this knowledge in order to design a drug that can achieve a specific goal. But this is seldom possible in the area of nutrition. This is because the action of foods and of nutrients and other food components in the body is typically of great complexity.

It must be stressed that even where mechanistic research fails to lead to advances that are of practical value in the prevention or treatment of disease, the new knowledge generated is still of much value as it provides a deeper understanding of the natural world, much like research in astronomy.

Another conclusion from the evidence presented here is that the combined use of observational studies (mainly prospective cohort studies) and RCTs is a far superior strategy than is mechanistic research in generating information of practical value in showing which dietary changes are most effective in the prevention of various diseases. A strong argument that confirms this is as follows. A great many reviews have been carried out into the relationship between a wide variety of dietary factors and the incidence or prevalence of various disorders. As far as this author is aware, the large majority of these reviews focus on the findings from cohort studies and RCTs while paying little attention to the findings from mechanistic research. Despite the abundance of evidence that mechanistic research is of limited value in the area of nutrition, only a handful of researchers have made this argument [e.g., (1)].

There are few signs that the lessons from the past have been learned. Of particular note, mechanistic research continues to receive a substantial proportion of research dollars. This is illustrated by gene-based personalized nutrition. This area of nutrition research has emerged in recent years. The goal is to integrate an individual's genetic, phenotypic, and health-related information to provide precise dietary guidance to improve health (37, 38). Based on the evidence presented in this paper it is very doubtful if this new development in mechanistic research will live up to the hype. Nevertheless, it now attracts wide interest.

## Data availability statement

The original contributions presented in the study are included in the article, further inquiries can be directed to the corresponding author.

## Author contributions

The author confirms being the sole contributor of this work and has approved it for publication.
